# Exploring the Intersection of Diabetes and Musculoskeletal Health

**DOI:** 10.31661/gmj.v14i.3884

**Published:** 2025-11-05

**Authors:** Morteza Nakhaei Amroodi, Khatere Mokhtari, Pouria Tabrizian

**Affiliations:** ^1^ Bone and Joint Reconstruction Research Center, Shafa Orthopedic Hospital, Department of Orthopedic, School of Medicine, Iran University of Medical Sciences, Tehran, Iran; ^2^ Department of Cellular and Molecular Biology and Microbiology, Faculty of Biological Science and Technology, University of Isfahan, Isfahan, Iran

**Keywords:** Diabetes, Orthopedic, Rotator Cuff Tear, Muscle Atrophy, Frozen Shoulder, Osteoarthritis, AGEs, Achilles Tendons, Tendon Healing, Rheumatoid Arthritis

## Abstract

Diabetes presents a significant health challenge worldwide, with profound implications extending beyond glycemic control to impact various bodily systems. This review explores the intricate relationship between diabetes and musculoskeletal disorders, shedding light on their epidemiology, pathophysiology, and clinical implications. Individuals with diabetes face a heightened risk of developing musculoskeletal conditions, particularly tendon disorders such as adhesive capsulitis rozen shoulder, rotator cuff tears, muscle atrophy, osteoarthritis and diabetic hand syndrome. Mechanisms underlying these disorders include inflammation, glycation, and impaired tendon homeostasis, exacerbated by factors like insulin resistance and oxidative stress. Furthermore, diabetes poses challenges in orthopedic surgery, leading to increased rates of surgical complications and poorer outcomes. Understanding the interplay between diabetes and musculoskeletal health is crucial for developing targeted interventions aimed at optimizing patient care and outcomes in this population.

## Introduction

Type 2 diabetes mellitus (T2DM) is a chronic condition characterized by sustained elevation of blood glucose levels [1]. Neglecting to address diabetes can result in substantial long-term complications that impact both the vascular and nervous systems [2]. Individuals with diabetes are at three times greater risk for developing all musculoskeletal disorders. However, they are especially prone to tendon conditions, which tend to be more resistant to treatment compared to those in non-diabetic patients [3][4]. Up to 50% of individuals who discontinue exercise programs for type 2 diabetes cite musculoskeletal symptoms as the reason [3]. A significant proportion of these cases are attributable to tendinopathy[4][5][6].

Type 2 diabetes can indeed induce immediate damage to various bodily systems upon its onset[7][8]. Interestingly, individuals with diabetes mellitus report twice the amount of musculoskeletal complaints compared to age and gender-matched healthy controls [9]. Despite its heightened prevalence and consequential social impact, musculoskeletal disorders remain comparatively understudied in relation to other complications associated with diabetes [10]. Risk factors for musculoskeletal disorders in individuals with diabetes include advanced age, longer duration of diabetes mellitus, and hypertension [11]. One example illustrates that more than 25% of individuals diagnosed with diabetes mellitus experience shoulder issues, with the prevalence of frozen shoulder ranging from 10% to 35% [12][13][14]. The most commonly reported shoulder issue in individuals with diabetes mellitus is frozen shoulder, clinically known as adhesive capsulitis [14].

While the precise cause of musculoskeletal disorders, including adhesive capsulitis, remains largely uncertain, several proposed pathogenic factors may contribute to their development in individuals with diabetes mellitus. These factors include the accumulation of irreversible crosslinks between neighboring protein molecules, vascular and neural damage, and elevated collagen levels in connective tissue [12]. Animal studies have provided evidence supporting the proposed pathogenic pathway underlying shoulder dysfunction in diabetes mellitus. Specifically, these studies have shown an increase in tendon diameter and stiffness in diabetic mice, suggesting a potential mechanism for the development of shoulder complications in individuals with DM [15].

Inflammation stands as the central mechanism driving tendon dysfunction in diabetes mellitus. Persistent secretion of inflammatory agents like TNF-α and IL-6 among diabetic individuals initiates a series of inflammatory processes. This prolonged inflammatory state prompts the buildup of collagen and other extracellular matrix elements, culminating in fibrosis and subsequent impairment of tendon function [16][17][18][19].

For example, Type 1 diabetes represents the primary risk factor for frozen shoulder, with incidence rates possibly reaching 59% in individuals aged over 45, and a lifetime prevalence of 76%. Patients with type 1 diabetes often experience more pronounced disability and a greater reduction in their range of motion compared to other groups [20]. The cumulative level of glycated hemoglobin A1c (HbA1c) serves as a significant determining factor, with patients exhibiting poorer blood glucose control facing an elevated risk for developing frozen shoulder [21]. Individuals with diabetes may indeed experience more adverse outcomes from frozen shoulder compared to those without diabetes. If high-quality studies can confirm the findings of this review, it underscores the importance for clinicians to carefully monitor diabetic patients with frozen shoulder and contemplate additional treatment if persistent pain or functional limitations persist in the long term [22].

## The Link Between Diabetes and the Musculoskeletal System

### 1.1. Diabetes in Orthopedic

Diabetes mellitus has been linked to unfavorable outcomes across various orthopedic surgery specialties. It’s crucial for orthopedic surgeons to prioritize enhancing preoperative, perioperative, and postoperative medical care in patients with diabetes mellitus. Elevated incidences of surgical site infections (SSIs) have been particularly observed in procedures such as total joint arthroplasty, spinal surgery, and foot and ankle operations.

Additionally, individuals with diabetes are more prone to developing other postoperative complications, including myocardial infarction, pulmonary embolism, and urinary tract infections.

They also tend to endure prolonged hospital stays and more non-routine discharges compared to non-diabetic counterparts.

Recent investigations indicate that diabetes mellitus itself may not be solely accountable for adverse outcomes. Instead, it is more likely that diabetes-related complications—such as poor glycemic control, neuropathy, end-stage renal disease, and peripheral artery disease (PAD)—contribute to the increased risk of adverse outcomes. In contrast, patients with well-controlled, uncomplicated diabetes mellitus typically experience outcomes comparable to those of individuals without diabetes [23].

**Figure-1:**
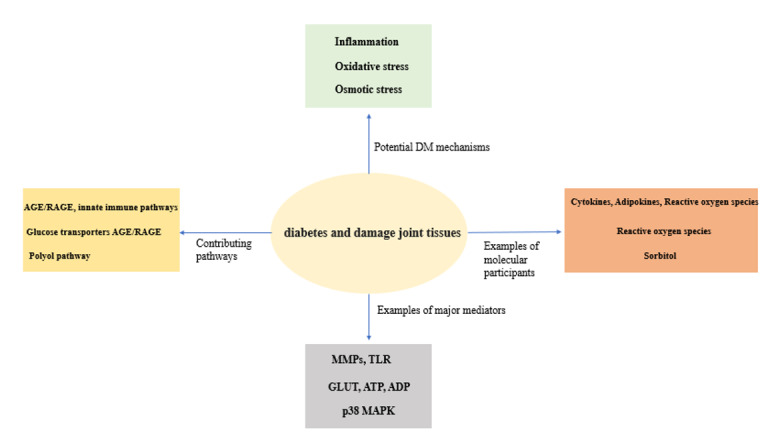
Figure 1. Mechanisms present in diabetes that can damage joint tissues (AGE/RAGE: Advanced Glycation End-products / Receptor for Advanced Glycation End-products, GLUT: Glucose Transporters, TLR: Toll-like Receptor, ATP: Adenosine Triphosphate, ADP: Adenosine Diphosphate, =MMPs: Matrix Metalloproteinases, p38 MAPK: p38 Mitogen-Activated Protein Kinase)

Basic science investigations have uncovered several potential mechanisms associated with joint damage influenced by DM [24][25][26][27][28][29][30] (Figure-1, Table-1).

### 1.2. Glycation and Tendon Mechanical Behavior

**Figure-2:**
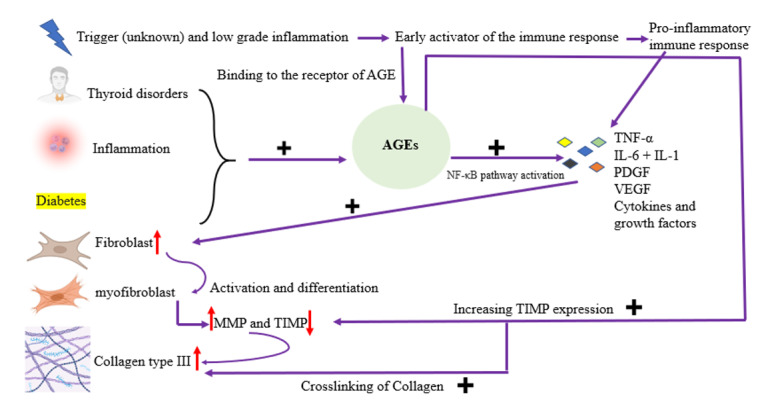
Figure 2. Diabetes and AGEs (TNF-α: Tumor Necrosis Factor alphaIL-1: Interleukin-1, IL-6: Interleukin-6, PDGF: Platelet-Derived Growth Factor, VEGF: Vascular Endothelial Growth Factor, MMP: Matrix Metalloproteinase, TIMP: Tissue Inhibitor of Metalloproteinases, NF-κB: Nuclear Factor kappa-light-chain-enhancer of activated B cells, AGE: Advanced Glycation End-products) The figure was generated using BioRender.

One of the primary factors contributing to tissue dysfunction in patients with diabetes is the increased glycation of proteins and the formation of Advanced Glycation End Products (AGEs) in their collagenous tissues [53][54][55][56]. AGEs represent a diverse array of compounds resulting from a non-enzymatic interaction between reducing sugars and the unbound amino groups present in proteins and lipids. This chemical process is referred to as the Maillard reaction [57][58][59][60]. Within a collagen-rich extracellular matrix, AGEs have the capacity to create crosslinks among collagen fibrils. These crosslinks subsequently influence various aspects including biomechanical characteristics, resistance to thermal fluctuations, susceptibility to enzymatic breakdown, and the arrangement of collagen molecules. Notably, AGE-mediated crosslinks endure for the entire lifespan of the associated protein, posing a significant issue particularly in tendon tissue where collagen turnover occurs at a comparatively gradual pace [61][62][63][64][65][66]. Tendons exhibit a hierarchical organization wherein collagen molecules align parallelly to construct fibrils. These fibrils further aggregate to create fibers, which in turn assemble into fascicles. Finally, these fascicles amalgamate to constitute the entirety of the tendon structure [67]. A significant characteristic of tendons is their capacity to reduce the strain encountered by each substructure relative to the larger structure along the length scale, termed strain attenuation. This property allows tendons to mitigate the accumulation of microdamage and enhance the maximum strain they can withstand before reaching failure [68][69]. Because of diminished collagen sliding, tendons affected by glycation often demonstrate reduced strain attenuation. This results in elevated strain exerted on individual fibers and fibrils while diminishing the maximum strain tolerance of the entire tendon. Consequently, this scenario has the potential to escalate microdamage at these finer length scales during routine tendon loading [70][71] (Figure-2). While this section focuses primarily on tendons due to their well-characterized hierarchical collagen structure and relatively low turnover rate, it is important to note that glycation-induced modifications also affect other musculoskeletal tissues. For instance, in articular cartilage, AGEs can impair proteoglycan content and disrupt collagen architecture, leading to increased stiffness and decreased shock absorption capacity [72][73]. In skeletal muscle, glycation may reduce contractile efficiency and regenerative capacity [74][75]. Similarly, ligaments and bone exhibit AGE accumulation that compromises biomechanical resilience, increases brittleness, and contributes to diabetic musculoskeletal fragility [76][77]. Therefore, glycation broadly affects multiple components of the musculoskeletal system, though tendons are particularly vulnerable due to their structural characteristics and metabolic profile.

### 1.3. Hyperglycemia and Tendon Cell Behavior

Diabetes mellitus exerts an influence on the functional and mechanical properties of tendons, which is mirrored in changes to the cellular milieu. The predominant cells in tendons, namely tenocytes and tendon stem/progenitor cells (TSPCs), assume crucial roles in maintaining tendon homeostasis, facilitating remodeling, and orchestrating repair processes [78]. A hyperglycemic environment and diabetic conditions can adversely affect tendon cells, leading to structural and functional alterations in diabetic patients’ tendons. These changes accelerate the progression of tendinopathy. Tenocytes serve as key cellular constituents of tendons, primarily responsible for remodeling extra cellular matrix (ECM) and preserving tissue function. They accomplish this by synthesizing collagen, proteins, and proteoglycans, which facilitate ECM remodeling and repair processes [79][80]. Multiple in vitro studies have demonstrated that tenocytes exposed to high-glucose conditions exhibit decreased proliferation and migration, accompanied by an increase in apoptotic activity [81][82][83][84]. Hyperglycemic conditions have been shown to facilitate the accumulation of AGEs [85][86]. Indeed, AGEs exert their effects on multiple cell types by binding to the receptor for AGEs (RAGE), thereby activating a range of intracellular signaling pathways [87][88][89]. Activation of AGE-RAGE can trigger apoptosis, modulate the expression of pro-inflammatory markers, and instigate degradation of ECM [90][91][92][93][94][95][96][97]. The disruption of tenocyte signaling linked to diabetes is thought to impact nearly all components of the extracellular matrix (ECM). While type I collagen predominantly constitutes tendon ECM, other ECM constituents like elastin and proteoglycans may also hold significant roles in tendon function [84][98].

Studies collectively highlight the significant impact of high glucose concentrations and AGEs on matrix organization and turnover within tendons. Notably, the precise levels of glucose that directly affect the tendon remain unclear, indicating a need for further investigation in this area [99]. Further in vivo research is essential to accurately quantify glucose concentrations within the tendon microenvironment across different stages of hyperglycemia. Additionally, there is a substantial lack of evidence regarding the therapeutic potential of insulin administration and antiglycation agents in alleviating hyperglycemia-induced damage. Notably, hyperglycemic conditions not only impair the expression of tendon-specific genes in tenocytes but also enhance the activation of adipogenic transcription factors, including PPARγ and C/EBPs [83].

The adipogenic transdifferentiation of tenocytes induced by high glucose levels could potentially facilitate the accumulation of lipid deposits within the tissue, exacerbating the deterioration of functional and biomechanical properties in tendons of diabetic patients [100]. Indeed, apart from tenocytes, a distinct niche population of TSPCs has been identified across various species [101]. TSPCs exhibit stem cell properties and hold substantial importance in the processes of tendon repair and regeneration [102][103]. Diabetes-related alterations in TSPCs are associated with either enhanced transdifferentiation or impaired regenerative and reparative capacity. Compared to their healthy counterparts, diabetic TSPCs exhibit reduced expression of CD44, a glycoprotein critical for regulating cell proliferation, survival, differentiation, and motility [104][105][106][107][108].

**Figure-3:**
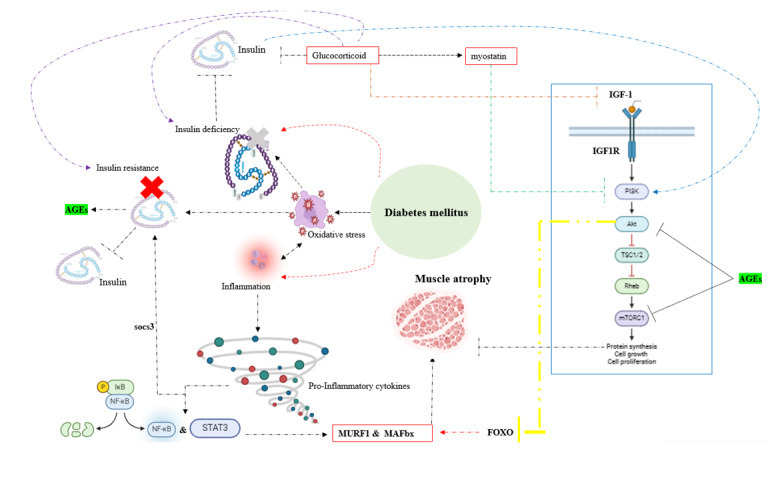
Figure 3. Key pathways involved in Diabetic muscular atrophy (AGEs – Advanced Glycation End Products, AKT – Protein Kinase B, FoxO – Forkhead Box O, IGF-1 – Insulin-like Growth Factor 1, IGF1R – Insulin-like Growth Factor 1 Receptor, IKB – Inhibitor of Nuclear Factor Kappa B, MAFbx – Muscle Atrophy F-box Protein (also known as Atrogin-1), mTORC1 – Mechanistic Target of Rapamycin Complex 1, MURF1 – Muscle RING Finger Protein 1, NF-κB – Nuclear Factor Kappa-light-chain-enhancer of Activated B Cells, PI3K – Phosphoinositide 3-Kinase, Rheb – Ras Homolog Enriched in Brain, SOCS3 – Suppressor of Cytokine Signaling 3, STAT3 – Signal Transducer and Activator of Transcription 3, TSC1/2 – Tuberous Sclerosis Complex 1 and 2) The figure was generated using BioRender.

## 2. Diabetic-Related Tendon Disorders

**Table:** Table1. Orthopedic and Diabetes

**Orthopedic fields related to diabetes **	**Description **	**Ref.s**
Disturbances of gait	People with diabetes may encounter restricted movement in the foot and ankle due to several factors including neuropathy, joint stiffness, and alterations in soft tissues. This constraint can impair mobility and increase the likelihood of developing foot deformities and sustaining injuries.	[31]
balance and stability	High glucose levels in individuals with diabetes can lead to thickening of the Achilles tendon and plantar fascia, contributing to conditions like Achilles tendinopathy and plantar fasciitis.	[32][33]
soft tissues tendon healing	High concentrations of glucose can adversely affect proteoglycan synthesis by tenocytes, contributing to tendon dysfunction and potentially impairing the healing process in individuals with diabetes.	[34][35]
Bone healing and metabolism	Bone mineral alterations are also recognized complications of diabetes mellitus. Individuals with diabetes may experience changes in bone density and metabolism, leading to an increased risk of osteoporosis and fractures.	[36][37][38]
Foot and ankle surgery	Neuropathy and peripheral artery disease increase the risk of infection, amputation, and complications such as Charcot neuroarthropathy (CN) in patients with diabetes mellitus. Additionally, diabetes can also predispose individuals to conditions like ankle fractures.	[39][40][41][42][43]
Postoperative complications of orthopedic surgery	Postoperative infections, cardiac complications	[44][45][46][47]
Sports medicine	Claudication	[48]
Total joint arthroplasty	Deep infection following primary total knee arthroplasty is a serious complication that can lead to significant morbidity and require extensive treatment.	[49]
Pediatric orthopaedics	Patients with diabetes mellitus may also be at risk of developing conditions such as slipped capital femoral epiphysis and tibia vara.	[50]
Upper extremity	Individuals diagnosed with diabetes mellitus are also at an increased risk of developing conditions like carpal tunnel syndrome, Dupuytren's disease, trigger finger, and limited joint mobility.	[51]
Spine surgery	In addition to experiencing heightened rates of surgical site infections and other complications, individuals with diabetes mellitus also encounter elevated rates of non-routine discharges, prolonged hospital stays, an increased need for blood transfusions, and higher hospital charges compared to those without diabetes mellitus.	[52]

### 2.1. Frozen Shoulder and Diabetes

Adhesive capsulitis of the shoulder, often known as frozen shoulder, presents as a painful and debilitating condition characterized by discomfort during abrupt movements and limited range of motion, notably in external rotation of the shoulder. Owing to its symptoms, frozen shoulder is often misdiagnosed. Managing frozen shoulder, especially in diabetic individuals, presents challenges, prompting clinicians to often favor one or two treatment modalities based on patient-specific factors and the severity of symptoms [109]. The incidence of FS typically ranges between 3 and 5%, but in diabetic patients, it can increase significantly, reaching up to 30%. Moreover, diabetic individuals with FS often experience more severe symptoms and may exhibit resistance to treatment interventions [110][111][112]. Frozen shoulder most commonly affects individuals in middle age, and it tends to impact women slightly more than men. Additionally, it can occur bilaterally, affecting both shoulders simultaneously or sequentially [112][113][114]. Frozen shoulder can develop as a secondary condition to trauma and is also linked with other connective tissue disorders such as Dupuytren’s contracture and Peyronie’s disease [115].

The exact pathophysiology of frozen shoulder remains incompletely elucidated; however, it is widely recognized that chronic inflammation contributes to the development of proliferative fibrosis. Gross anatomical observations commonly reveal capsular thickening and vascular congestion, with prominent inflammatory changes particularly localized to the rotator interval, the coracohumeral ligament, and the middle glenohumeral ligament [116]. Microscopic examination of the affected capsule in frozen shoulder shows an augmented presence of fibroblasts, mast cells, macrophages, and T cells [117]. Synovitis is linked to increased levels of fibrotic growth factors, inflammatory cytokines, and interleukins, contributing to the pathogenesis of frozen shoulder [116].

To explain the increased occurrence of frozen shoulder in individuals with diabetes mellitus, it has been proposed that elevated systemic glucose levels accelerate glycosylation. This mechanism could contribute to higher rates of frozen shoulder and other soft tissue disorders, such as Dupuytren’s disease [118]. A correlation exists between elevated levels of HbA1c and the onset of frozen shoulder in diabetic patients [21]. Arthroscopic synovial tissue biopsies from diabetic patients with frozen shoulder have demonstrated elevated levels of endothelial growth factors in comparison to non-diabetic individuals with the same condition [119] Moreover, diabetic patients display decreased levels of inflammatory growth factors, such as ADAMTS-4, MMP-1, and notably M-CSF [120]. The decrease in inflammatory growth factors, especially M-CSF, might contribute to a slowed inflammatory response, potentially prolonging and intensifying the severity of the disease. Nevertheless, some studies have reported minimal discrepancies in inflammatory markers between diabetic and non-diabetic patients [121]. In other word There is a direct correlation between the cumulative hemoglobin A1c level and the incidence of frozen shoulder [21]. Frozen shoulder tends to persist for longer periods and is more resistant to conservative treatments in diabetic patients [122].

The precise mechanism behind this phenomenon is likely multifaceted. Some researchers have postulated that AGEs play a significant role. AGEs form through non-enzymatic glycation, a process in which glucose chemically binds to proteins, induced by oxidative stress. Once formed, AGEs form stable bonds with long-lived proteins, impeding their normal turnover and leading to their accumulation in connective tissues. While this process is a natural part of aging and can be mitigated by endurance training, it is accelerated in individuals with diabetes mellitus [123]. One particular non-enzymatic reaction of interest involves the glycation of collagen proteins, leading to the formation of crosslinks [124]. Increased levels of advanced glycation end products can trigger pathological collagen crosslinking, thereby modifying tissue structure and diminishing its compliance [125]. Capsular tissue samples taken from patients with frozen shoulder have shown notably elevated levels of advanced glycation end products compared to control samples [126]. AGEs have been shown to reduce the expression of matrix metalloproteinases and increase the expression of TIMP in diabetic nephropathy. This imbalance in ECM turnover mirrors the pathogenic mechanism observed in frozen shoulder [127].

Moreover, in diabetic retinopathy and nephropathy, the accumulation of advanced glycation end products has been demonstrated to enhance the expression of basic fibroblast growth factor and upregulate the expression of profibrotic cytokines such as TGF-β1, PDGF, and connective tissue growth factors [128]. It is hypothesized that these pro-fibrotic actions of AGEs also play a role in the pathophysiology of frozen shoulder, potentially elucidating why frozen shoulder in diabetic patients often shows resistance to treatment [126].

### 2.2. Diabetic Hand Syndrome

Diabetic hand syndrome (DHS) encompasses several distinct conditions, including limited joint mobility (LJM) or diabetic cheiroarthropathy, Dupuytren’s disease (DD), and flexor tenosynovitis/trigger finger (FTS). Regardless of the particular pathology present, DHS is commonly characterized by a positive prayer sign, where patients are unable to fully approximate their fingers and palms [129]. Diabetic cheiroarthropathy is the most common manifestation of DHS, with reported prevalence rates ranging from 20% to 54% in individuals with T2DM [130][131]. LJM is characterized by hand stiffness resulting from flexion contractures of the fingers. This condition affects not only the flexor tendons but also extends to the synovial sheath and surrounding subcutaneous tissues [132]. Dupuytren’s disease is characterized by fibrosis of the palmar fascia, resulting in the development of flexion contractures of the digits. Its prevalence ranges between 14% to 63% [133][134]. FTS constitutes the third most common component of DHS, with an incidence ranging from 11% to 20%. This condition manifests as the "locking" of the finger during flexion. While the first, third, and fourth digits are most frequently affected, diabetic individuals are more prone to experiencing impairment in multiple fingers [132][134][135][136]. Indeed, the coexistence of multiple conditions within DHS can exacerbate functional deficits in affected individuals [131]. Although Carpal Tunnel Syndrome (CTS) is classified as a compression neuropathy rather than a component of the diabetic hand syndrome per se, it is frequently discussed in the context of diabetic hand conditions due to its high prevalence in diabetic populations. CTS arises from compression of the median nerve by the transverse carpal ligament and represents one of the most common pathological conditions affecting the diabetic hand. The prevalence of CTS in diabetic patients has been reported to range between 14% and 60% [132][137][138][139].

### 2.3. Achilles Tendons and Diabetes

The structural and functional alterations in the tendons of the feet can significantly impact daily activities and may result in changes in gait and loading patterns, thereby increasing the risk of diabetic foot ulcers. The study examined alterations in foot function with a specific emphasis on the Achilles tendon (AT)-plantar fascia-metatarsophalangeal joint complex. Findings revealed significant thickening of both the Achilles tendon and plantar fascia in diabetic patients. Furthermore, joint mobility was substantially reduced, accompanied by notable changes in loading patterns. These findings underscore the importance of monitoring foot function in diabetic patients to mitigate the risk of diabetic foot complications [140].

The collective impact of these changes can indeed alter gait and loading patterns in patients with T2DM. Foot ulcers, a common complication in T2DM, are believed to be associated with increased passive stiffness of the muscle-tendon unit. Batista et al., utilizing ultrasound, illustrated a notable increase in the prevalence of tendon fiber disorganization in the Achilles tendon, with 89% of T2DM patients affected compared to only 10% of non-diabetic controls [141]. That’s an intriguing finding. Abate et al. demonstrated that asymptomatic T2DM patients exhibited a heightened incidence of ultrasound abnormalities in the AT compared to non-diabetic individuals [142]. This observation suggests that a significant number of diabetic patients likely harbor degenerative tendon changes that have not yet manifested clinically [143]. These findings underscore the pervasive nature of tendon pathology in T2DM patients, emphasizing the need for comprehensive evaluation and management strategies in this population.

### 2.4. Rotator Cuffs and Diabetes

Tears of the rotator cuff (RC) have been inherited from our ancestors and are associated with the great apes [144][145]. With the advent of newer techniques, patients who are appropriately selected and compliant can anticipate achieving good to excellent results [146][147]. Indeed, numerous reports and epidemiological studies have underscored the potential association between diabetes mellitus and tendon alterations in different anatomical regions of the body [148][149]. Diabetes negatively impacts the mechanical properties of native tendons and the healing process of injured tendons [150]. Extended periods of hyperglycemia heighten the probability of anatomical failure in the rehabilitated rotator cuff. Additionally, diabetes mellitus has been recognized as an independent risk factor for the development of rotator cuff disease, indicating that individuals with diabetes are more susceptible to experiencing tears in the rotator cuff. [151][152].

Although no preoperative factor definitively predicts setbacks, it’s worth noting that setbacks are often linked with inferior clinical outcomes compared to successful repairs. However, among various comorbidities like smoking, obesity, high blood cholesterol, and age, diabetes notably impacts the recovery rate, resulting in earlier plateaus and overall poorer outcomes [153][154][155][156]. Cumulative evidence suggests that individuals with diabetes generally have worse structural and functional outcomes after rotator cuff surgery compared to those without diabetes. However, several studies have shown no significant differences in clinical scores between diabetic and non-diabetic patients at the final follow-up [157][158][159]. Moreover, a study demonstrated that the mean enhancement in pre- and post-operative outcome scores was notably higher in non-diabetic patients compared to diabetic patients. This implies that the influence of diabetes on outcome scores remains uncertain. Additionally, recent studies have explored preoperative clinical factors predicting arthroscopic rotator cuff repair’s success and have indicated that diabetes is not a predictor of rotator cuff laxity [159][160][161][162][163][164]. To date, the impacts of diabetes on outcomes following rotator cuff repair and the influence of sustained hyperglycemia on retraction rates have not been fully characterized.

### 2.5. Diabetic Tendon Healing

It is evident that type 2 diabetes mellitus (T2DM) disrupts tendon homeostasis and baseline function while also markedly impairing the healing response after tendon injury and surgical repair. Although physiological tendon healing may occasionally result in suboptimal outcomes, the presence of T2DM further aggravates this process, increasing the propensity for fibrotic healing of the tendon. This underscores the challenges clinicians face in managing tendon injuries in diabetic patients and emphasizes the importance of tailored approaches to optimize healing outcomes in this population [165].

Absolutely, the increased risk of tendon tear or rupture by up to five-fold in individuals with T2DM compared to non-diabetics underscores the critical importance of addressing tendon health in diabetic patients. This heightened vulnerability to tendon injuries necessitates proactive management strategies aimed at optimizing tendon health, preventing injuries, and facilitating optimal healing outcomes in diabetic individuals [166]. The rotator cuff indeed has garnered the most abundant clinical data regarding healing outcomes in specific tendons. The evidence consistently demonstrates diminished healing and a heightened risk of repair failure, with the risk being more than two-fold greater in individuals with T2DM compared to non-diabetic counterparts.

These findings highlight the critical importance of carefully managing rotator cuff injuries in diabetic patients to optimize healing outcomes and minimize the risk of repair failure [157][167]. Indeed, limitations in tendon healing appear to be particularly pronounced during the early phases of the healing process. Clement et al. demonstrated that although improvements in pain and function were observed in T2DM patients at the 6-month postoperative mark, the magnitude of these improvements was markedly reduced compared to non-diabetic patients. These findings underscore the importance of closely monitoring and managing diabetic patients during the critical early phases of tendon healing to optimize outcomes and mitigate the impact of T2DM on the healing process [158].

Hsu et al.’s findings provide an interesting contrast, as they identified no difference in outcomes between diabetic and non-diabetic patients in the long term, specifically beyond 24 months postoperatively. This suggests that while there may be initial differences in healing outcomes between diabetic and non-diabetic individuals, these disparities may diminish over time. It’s important for clinicians to consider both short-term and long-term outcomes when managing tendon injuries in diabetic patients, recognizing the potential for variability in healing trajectories over time [168].

## 3. Diabetic Muscle Atrophy and Its Mechanisms

**Table:** Table2. Drugs for Diabetic Muscular Atrophy

**Drugs**		**Effect on muscle**		**Ref.s**
	Attenuate the muscle wasting	repair	Against Muscle atrophy	
Thiazolidinedione	*			[240]
Metformin		*		[240][241][242]
Vitamin D		*		[243][244]
Omega-3 fatty acid			*	[245][246][247][248]
Insulin		*		[240]

### 3.1. General Overview of Muscle Atrophy in Diabetes

Disturbances in the primary pathways of protein degradation and synthesis are implicated in muscle atrophy. Key pathways involved in protein synthesis, such as the insulin-like growth factor-1-phosphoinositide-3-kinase-Akt/protein kinase B-mammalian target of rapamycin (IGF1-PI3K-Akt/PKB-mTOR) pathway and the IGF-1-AKT-FoxO pathways, are pivotal in this context. Dysfunctions within these pathways can result in muscle wasting and atrophy [169][170][171][172][173][174][175][176]. In type 2 diabetes, insulin resistance suppresses the IGF-1-PI3K-AKT/PKB-mTOR pathway, leading to inhibition of protein synthesis. Moreover, insulin resistance contributes to muscle atrophy by stimulating the ubiquitin-proteasome system and the autophagy-lysosome pathway through the IGF-1-AKT-FoxO signaling pathway. Conversely, in type 1 diabetes, muscle atrophy is frequently mediated by a protein degradation pathway based on FoxO [177][178][179][180]. Additionally, muscle atrophy in individuals with diabetes can also be attributed to damage caused by oxidative stress, inflammatory responses, and elevated levels of glucocorticoids [181][182] (Figure-3).

### 3.2 Role of Insulin Resistance in Diabetic Muscular Atrophy

Muscle contraction relies significantly on insulin-stimulated glucose uptake. Insulin serves as a potent synthetic signal that greatly enhances muscle protein synthesis [183][184]. Activation of PI3K, PDK1, AKT, mTOR, p70S6K pathways leads to the phosphorylation and activation of downstream targets involved in protein synthesis, ultimately promoting muscle hypertrophy and growth. These pathways represent key targets for interventions aimed at enhancing muscle mass and function, particularly in conditions associated with muscle wasting or impaired muscle growth [185][186]. Glucose serves as a primary fuel source for muscle activity during contraction, and its availability is tightly regulated by insulin. When insulin signaling is disrupted, as in DM, skeletal muscle may experience inadequate glucose uptake, leading to compromised muscle contraction and function. This impairment in glucose utilization contributes to the muscle weakness and decreased exercise capacity often observed in individuals with DM [187].

maintaining proper insulin sensitivity and signaling is crucial for preserving muscle mass and function in individuals with DM [188]. Sarcopenia is indeed a recognized complication of T2DM. It’s characterized by the gradual and progressive loss of skeletal muscle mass and function, leading to reduced strength, mobility, and overall physical performance. The interplay of various factors, including insulin resistance, chronic inflammation, hormonal imbalances, and impaired protein metabolism, contributes to the development and progression of sarcopenia in individuals with T2DM. Managing blood glucose levels, promoting physical activity, and optimizing nutritional intake are crucial strategies for mitigating the risk of sarcopenia and preserving muscle health in individuals with T2DM [189][190][191]. In the context of insulin resistance, signaling pathways mediated by insulin or IGF-1 are inhibited, resulting in the suppression of the PI3K/AKT pathway. This suppression leads to decreased mTOR activity and a subsequent reduction in protein synthesis, which collectively contribute to muscle atrophy observed in patients with type 2 diabetes mellitus [192]. Moreover, insulin resistance results in elevated systemic glucose levels, facilitating the interaction of glucose with proteins or lipids, leading to the formation of AGEs [193]. AGEs play a pivotal role in the development of chronic diabetic complications. Additionally, the buildup of AGEs is considered a potential contributor to muscle loss and weakness in individuals with T2DM [194].

The receptor for advanced glycation end products (RAGE) is a transmembrane signaling receptor implicated in the development of diabetic renal and vascular complications. Activation of RAGE by AGEs can promote muscle atrophy and impair myogenesis by inhibiting AKT signaling through the activation of AMPK pathways [195]. Furthermore, AGEs have been shown to interfere with muscle anabolic signaling by suppressing the mTORC1 pathway [194]. In summary, insulin resistance can impair the IGF-1-PI3K-AKT-mTOR signaling pathway responsible for protein synthesis, leading to reduced protein production and subsequent skeletal muscle atrophy.

### 3.3. Role of Insulin Deficiency in Diabetic Muscular Atrophy

Individuals with T1DM demonstrate diminished repair capacity in their skeletal muscle satellite cells and experience skeletal muscle dysfunction. These abnormal phenotypes are associated with insulin deficiency, which disrupts the balance between protein degradation and synthesis, leading to degradation rates that surpass synthesis rates [196]. Under physiological conditions, both the insulin receptor (IR) and the IGF-1 receptor (IGF-1R) regulate multiple cellular functions through the PI3K/AKT signaling pathway. For example, during glucose uptake and protein synthesis, AKT activation triggered by insulin or IGF-1 results in the phosphorylation of FoxO transcription factors, thereby suppressing their transcriptional activity [197]. In insulin-deficient diabetes or conditions characterized by impaired insulin/IGF-1 signaling in muscle, there is a decrease in complex I-driven mitochondrial respiration and supercomplex assembly. This effect is mediated by FoxO transcription factors, which suppress the expression of complex I subunits [198]. These effects have significant implications for mitochondrial function and contribute to the induction of skeletal muscle atrophy [197]. In summary, insulin deficiency leads to enhanced transcriptional activity of FoxO, which subsequently upregulates the expression of muscle atrophy-related genes, ultimately resulting in muscle atrophy.

### 3.4. Role of Inflammation in Diabetic Muscular Atrophy

IL-6 is a pro-inflammatory cytokine well-known for its impact on muscle tissue [199][200]. Individuals with T2DM frequently present with increased circulating levels of inflammatory markers such as C-reactive protein, IL-1β, and IL-6 [201]. In type 1 diabetes mellitus (T1DM), skeletal muscle regeneration is impaired due to dysfunction of satellite cells [202]. Therefore, persistently elevated IL-6 levels may play a role in satellite cell dysfunction associated with diabetes mellitus. Additionally, hyperglycemia can induce the release of inflammatory mediators, including IL-6, activate immune cells such as macrophages, and trigger apoptosis-related signaling pathways, notably the Fas/FasL pathway [203][204]. Indeed, this stimulation can contribute to islet β-cell dysfunction, resulting in insulin deficiency. Furthermore, IL-6 has been shown to promote insulin resistance by reducing insulin sensitivity and altering lipid metabolism [205][206]. Insulin mediates its biological effects by binding to the insulin receptor (IR). However, the pro-inflammatory cytokine TNF-α can interfere with the tyrosine phosphorylation and activation of IR within the insulin signaling pathway, thereby contributing to the development of insulin resistance [206].

Additionally, TNF-α can reduce glucose uptake and utilization in skeletal muscle and adipocytes by downregulating the expression of the glucose transporter GLUT4. This effect contributes to the development of insulin resistance and facilitates muscle atrophy [207][208].

STAT3 can be activated by pro-inflammatory cytokines such as IL-6, resulting in the suppression of signaling pathways involved in protein synthesis within muscle tissue. [209][210][211][212]. NF-κB serves as a central transcriptional regulator that induces the expression of a wide array of genes. Its activation can be triggered by various stimuli, including viral infections, TNF, and B cell activating factor (BAFF) [213][214]. Moreover, NF-κB can promote the degradation of certain muscle proteins by upregulating the expression of the E3 ubiquitin ligase MuRF1 [215][216][217]. The NF-κB and STAT3 signaling pathways function as key mediators of inflammation and can be significantly activated by elevated levels of pro-inflammatory cytokines, such as TNF-α, and non-esterified fatty acids. This activation results in the upregulation of MuRF1 expression, which in turn stimulates the ubiquitin-proteasome system (UPS), promoting muscle protein degradation [218][219]. Additionally, IL-6 may contribute to muscle atrophy by modulating the activity of IGF-1 [220][221].

### 3.5. Role of Oxidative Stress in Diabetic Muscular Atrophy

The elevated metabolic activity of skeletal muscle makes it especially susceptible to damage caused by oxidative stress [220]. Oxidative stress impairs the AKT-mTOR signaling pathway and its downstream effectors, thereby inhibiting protein synthesis and promoting muscle atrophy [222][223]. Moreover, islet β cells are highly vulnerable to ROS due to their inherently low levels of antioxidant enzymes. ROS can cause direct damage to β cells, leading to apoptosis, and can also indirectly disrupt insulin signaling pathways and impair β cell function, ultimately contributing to the development of diabetes mellitus [224][225]. ROS act as key mediators in the activation of pro-inflammatory signaling pathways [226][227]. A persistent inflammatory milieu fosters the generation of free radicals, including ROS. This exacerbates β-cell injury, establishing a positive feedback loop where additional detrimental cytokines are released, prompting further harm to β cells [228]. Oxidative stress can induce insulin deficiency and generate substantial quantities of ROS that impede insulin signaling transduction, consequently precipitating insulin resistance [229]. Ultimately, this sequence of events can contribute to the onset of skeletal muscle atrophy.

### 3.6. Role of Glucocorticoids in Diabetic Muscular Atrophy

Cortisol (GC) is a hypoglycemic hormone that stimulates gluconeogenesis and glycogen breakdown, thereby opposing the effects of insulin and elevating blood glucose levels [230]. GC signaling plays a significant role in contributing to muscle atrophy in diabetes mellitus [231]. Furthermore, upon binding to the glucocorticoid receptor (GR), GC inhibits AKT, GLUT4, and IR signaling, consequently inducing insulin resistance [232]. In cases of T1DM characterized by insulin deficiency, the presence of GCs alongside insulin deficiency in muscle prompts competition between GR and IRS1 for binding to PI3K subunits P110 and p85. Consequently, phosphorylation levels of IRS, PI3K, and AKT decrease, ultimately resulting in muscle atrophy [232].

GCs predominantly induce muscle atrophy by enhancing protein breakdown through the UPS and autophagy-lysosome pathway (ALP), while concurrently diminishing protein synthesis via inhibition of the IGF-1-PI3K-AKT-mTOR and mTOR/p70S6k pathways [233][234][235]. Moreover, GCs upregulate the production of myostatin, which in turn reduces protein synthesis by inhibiting the AKT-mTOR pathway [236]. GCs can cause muscle atrophy by binding to their receptors, disrupting the insulin/IGF-1 signaling pathway, and promoting the transcription of dystrophin. Additionally, GRs can work together with FoxO1 to induce MuRF1, further accelerating muscle atrophy [237]. Furthermore, GRs regulates muscle catabolism by influencing the expression of MAFbx and MuRF1 [238][239]. Indeed, GCs contribute to skeletal muscle atrophy through various pathways. That sounds like a comprehensive approach to understanding the effects of anti-diabetic drugs on muscle atrophy in diabetes (Table-2).

## 4. Diabetes and Joint Diseases

### 4.1. Osteoarthritis and Diabetes

Osteoarthritis (OA) is indeed becoming more prevalent and is a significant health concern affecting millions of people worldwide [249]. OA is commonly described as a degenerative process affecting the joints, characterized by the erosion of articular cartilage, changes in the bone beneath and around the cartilage, mild to moderate inflammation of the joint lining, and pain. While damage to cartilage is the primary feature of OA, abnormalities in other tissues like tendons, bones, or muscles may also contribute to or initiate the condition. There are notable similarities between DM, particularly T2DM, and OA in terms of their epidemiological characteristics. Both conditions are complex, exhibiting substantial clinical diversity and multifaceted causes involving interactions between genetic predisposition and environmental factors. They also share common risk factors, with aging being a notable one. In the US, the prevalence of diabetes mellitus is 3.3 cases per 1000 individuals aged 18-44, increasing to 15.4 cases per 1000 individuals aged 65-79 [250].

Likewise, the prevalence of OA substantially rises with age, impacting 13.5% of adults aged 25 years and older, and notably affecting 33.6% of individuals aged 65 and above [251]. Another significant risk factor for both conditions is obesity. The link between OA and obesity is well-established, and a majority of individuals with T2DM are also affected by obesity [252][253]. The co-occurrence of OA and DM often happens coincidentally due to their high prevalence and overlapping risk factors. Approximately 47.3% of individuals with DM have some manifestation of arthritis [254]. The existence of comorbid conditions generally amplifies the care requirements of individual patients, reduces the efficacy of treatment, and raises healthcare expenses. Moreover, treatment approaches that prioritize personalized medicine and consider comorbidities may lead to better outcomes for OA patients [255]. The onset of OA could also complicate DM. Although not the primary focus of this review, emerging evidence suggests that OA contributes to the cardiovascular disease burden, which is already elevated in DM patients [256]. There are a growing acknowledgment of the significant role inflammation plays in both osteoarthritis and diabetes mellitus, serving as a crucial mechanistic connection between these two conditions. OA is characterized by notable synovitis, which may be aggravated by elevated levels of inflammatory cytokines, adipokines, and prostaglandins observed in tissues affected by DM [257][258].

Signaling through innate immunity pathways, such as toll-like receptors, can also induce inflammation in both diabetes mellitus and osteoarthritis [259][260]. In a state of hyperglycemia, there is increased generation of reactive ROS, which play a role in tissue damage. The regulation of cellular glucose transport becomes critical and may worsen oxidative stress. Research indicates that chondrocytes from older donors (aged 66 years and above) with osteoarthritis, when exposed to high glucose environments, demonstrate an impaired ability to downregulate GLUT1 protein expression or decrease glucose transport activity compared to chondrocytes from younger donors (aged 28-35 years) without OA [261]. Furthermore, it was noted that under high glucose conditions, there was an inclination towards increased oxidant production and enhanced matrix catabolism, potentially hastening the progression of osteoarthritis. It's important to highlight that age and media osmolarity were not standardized in these experiments [262].

The impacts of elevated glucose levels may be associated with compromised functionality of ATP-sensitive potassium (K+) channels. These channels are involved in coupling GLUT with intracellular ATP/ADP levels and membrane potential [263][264]. The AGE/RAGE (advanced glycation end products/receptor for AGEs system is another factor contributing to end-organ damage in diabetes mellitus by promoting inflammation and/or exacerbating oxidative stress. Collagen, known for its notably slow turnover rate in numerous connective tissues, is especially vulnerable to modification by AGEs. The accumulation of AGEs is accelerated by heightened levels of glucose in the tissues [265]. AGEs exert their effects by signaling through RAGE (receptor for AGEs) and other receptors, resulting in various harmful effects on chondrocytes. These effects include inflammation and cytokine-mediated catabolism. AGEs have been implicated in contributing to end-organ damage in diabetes mellitus [265][266][267][268].

Moreover, AGE-mediated cross-linking of collagen can modify the biomechanical properties of tissues, as evidenced in studies involving cartilage and tendon [269]. Cross-linking facilitated by AGEs may additionally hinder extracellular matrix turnover by impeding access to proteolytic sites [268]. Conversely, a recent investigation conducted on dogs indicated that artificially elevating AGE levels alone through repeated ribose injections did not hasten osteoarthritis progression in a mild injury model (129). However, our understanding of the impact of AGEs within the diabetic context on OA development remains limited. Therefore, the extent to which AGEs contribute to OA pathogenesis remains uncertain [270]. In the polyol pathway, glucose is converted to sorbitol and galactose to galactitol by aldose reductase. This pathway becomes activated in diabetes mellitus, resulting in an accumulation of polyols, which in turn induces cellular osmotic stress [271].

While a direct link to osteoarthritis has not been established, there is evidence suggesting that this pathway becomes activated in diabetes mellitus within intervertebral disc cartilage. Its activation appears to enhance matrix catabolism via p38 MAPK activation [272]. Although not extensively discussed in this review, it is noteworthy that other pathways relevant to diabetes mellitus have been proposed. For instance, substantial evidence suggests that adipokines might trigger inflammation and exert detrimental effects on cartilage and tissue healing [273][274]. Further research is needed to explore the role of adipokines in osteoarthritis among obese patients with diabetes mellitus, as altered adipokine levels are observed in obesity regardless of diabetes status [275]. Changes in angiogenesis, autophagy, and apoptosis have also been linked to end-organ damage in osteoarthritis [276][277]. Chondrocytes express insulin receptors, implying that elevated insulin levels, as observed in patients with type 2 diabetes mellitus, could potentially harm cartilage. A study demonstrated downregulation of PPARγ in articular chondrocytes exposed to high glucose media, although further research is needed to validate this result due to methodological complexities [261][278]. Additional investigation is required to ascertain the particular pathways implicated, prioritize the most pertinent pathways, and elucidate how molecular mediators intersect across multiple pathways concerning joint damage associated with diabetes.

### 4.2. Rheumatoid Arthritis

Rheumatoid arthritis (RA) is an autoimmune and inflammatory condition marked by sustained inflammation of the synovium, leading to cartilage and underlying bone deterioration [279][280]. The systemic inflammation linked to RA may potentially elevate the likelihood of developing diabetes later on. Markers of ongoing inflammation, such as CRP, are correlated with a heightened risk of diabetes in individuals with RA. Additionally, other conventional risk factors for T2DM are notably prevalent among those with RA [281] and may contribute to the higher risk of diabetes [282]. Due to chronic joint pain, swelling, and stiffness, individuals with RA often experience physical inactivity. This reduced activity level contributes to the development of T2DM through decreased calorie expenditure [283]. Rheumatoid arthritis is linked to a heightened risk of developing diabetes mellitus. This observation reinforces the concept of inflammatory pathways playing a role in the development of diabetes. Therefore, it is advisable to contemplate more aggressive interventions aimed at managing diabetes risk factors in individuals with rheumatoid arthritis [280].

## Conclusion

In conclusion, the correlation between diabetes mellitus and orthopedic disorders, including frozen shoulder, rotator cuff tears, muscle atrophy, osteoarthritis, tendinopathy, Rheumatoid arthritis, and underscores the complex interplay between metabolic dysfunction and musculoskeletal health. Individuals with DM exhibit a heightened susceptibility to these orthopedic conditions, often experiencing more severe symptoms and poorer treatment outcomes compared to non-diabetic counterparts. The underlying pathophysiological mechanisms involve chronic inflammation, altered protein degradation, oxidative stress, and impaired tissue healing, collectively contributing to the development and progression of musculoskeletal complications in diabetic individuals. Orthopedic surgeons and healthcare providers must prioritize comprehensive preoperative, perioperative, and postoperative management strategies tailored to address the unique needs of diabetic patients. Optimizing glycemic control, managing comorbidities, and implementing multidisciplinary approaches are essential for mitigating the risk of adverse outcomes and improving the overall prognosis of orthopedic interventions in this patient population. Furthermore, continued research efforts are warranted to elucidate the intricate molecular pathways linking diabetes and orthopedic disorders, identify novel therapeutic targets, and develop personalized treatment modalities. By advancing our understanding of these pathogenic mechanisms, clinicians can enhance clinical decision-making, optimize treatment efficacy, and ultimately improve the quality of life for individuals living with diabetes and orthopedic comorbidities.

## Conflict of Interest

There is no conflict of interest.
